# EPR spectroscopy of complex biological iron–sulfur systems

**DOI:** 10.1007/s00775-018-1543-y

**Published:** 2018-02-21

**Authors:** Wilfred R. Hagen

**Affiliations:** Department of Biotechnology, Delft University of Technology, Van der Maasweg 9, 2629HZ Delft, The Netherlands

**Keywords:** Iron–sulfur, EPR, Systems biology

## Abstract

From the very first discovery of biological iron–sulfur clusters with EPR, the spectroscopy has been used to study not only purified proteins but also complex systems such as respiratory complexes, membrane particles and, later, whole cells. In recent times, the emphasis of iron–sulfur biochemistry has moved from characterization of individual proteins to the systems biology of iron–sulfur biosynthesis, regulation, degradation, and implications for human health. Although this move would suggest a blossoming of System-EPR as a specific, non-invasive monitor of Fe/S (dys)homeostasis in whole cells, a review of the literature reveals limited success possibly due to technical difficulties in adherence to EPR spectroscopic and biochemical standards. In an attempt to boost application of System-EPR the required boundary conditions and their practical applications are explicitly and comprehensively formulated.

## Introduction

EPR spectroscopy and iron–sulfur biochemistry have always formed two closely intertwined fields. In fact, iron–sulfur proteins gave their first sign of existence to us in the form of an EPR signal [1] with a *g* value (1.94, i.e., significantly less than 2) that was initially considered to be impossible for an iron complex. Eventually its nature was found to reflect superexchange between two or more clustered iron ions [2, 3], and this initiated an exciting research period into the multifaceted structure of Fe/S clusters with the identification of 2Fe, 3Fe, 4Fe, 8Fe, and mixed-metal clusters. Concomitantly, biological functions were identified, initially, the obvious transfer of electrons by, e.g., ferredoxins (with the not so obvious finding that redox steps always involve a single electron, perhaps even in the 8Fe P-cluster of nitrogenase [4]), followed by the non-redox Lewis-acid catalysis by, e.g., aconitase [5, 6], which in turn naturally (but only in hindsight) led to the research of regulation, e.g., of iron homeostasis by the cytoplasmic aconitase or iron-responsive element-binding protein [7, 8]. In addition, exponentially increasing research efforts are seen in the areas of (presumably) redox sensing Fe/S clusters associated with DNA replication and repair enzymes [9] and of intramolecular electron transfer in radical SAM enzymes [10]. In recent years, the field of biosynthesis of iron–sulfur holo-proteins has established prominence as a new direction of study [11, 12]. And scattered through the earlier history of iron–sulfur biochemical research we see an occasional speck [13] foreboding a recent development of medical iron–sulfur research in particular related to cellular disfunctioning [14–16].

Since the subject of medical science is (supra)cellular by nature, a link with iron–sulfur EPR may require samples of a complexity well beyond that of neat, purified proteins. This really brings us back full circle to the early days of biomolecular EPR, in which the spectroscopy, almost from the onset onwards, has been applied to systems of considerable complexity such as respiratory chains in mitochondria [17]. To be clear: complexity here is used in the sense of having many different parts but not necessarily in the sense of being fundamentally difficult to unravel. The implication for spectroscopy is twofold: we may have to deal with the practical problem of multiple signal overlap and, potentially even more seriously, we will have to deal with that of low concentration of individual iron–sulfur centers. Particularly facing this latter challenge will be the red thread of the present paper: there are two types of iron–sulfur EPR spectroscopy defined by the complexity (number of parts) of the subject of study. The spectroscopy of purified proteins and of synthetic model systems is not seriously hampered by concentration limits; it can, therefore, bloom into the many directions of advanced EPR, such as multi-frequency, double-resonance, high-field projects. On the other hand, the spectroscopy of complex systems deals with several orders of magnitude lower concentration, say, (sub-) micromolar instead of (sub-) millimolar, and this, as will be explained in more detail below, not only limits the EPR methodology to the basic continuous-wave X-band experiment, but in addition calls for specific precautions and approaches both in the setup of the measurements and in the analysis of the results. For brevity we will call this approach: System-EPR.

## Paramagnetism of iron–sulfur clusters

Both elements Fe and S have a rich redox chemistry ranging from Fe(−II) to Fe(VI) and from S(−II) to S(VI). With the biological limitations of water as the solvent and protein as the major ligand the oxidation states of Fe are limited to range from a rare Fe(I) in hydrogenase to Fe(+IV) in, e.g., monoxygenases, but possible spin states still cover the range from *S* = 0 to *S* = 5/2 [including the quantum spin mixture 3/2–5/2 for some Fe(III) proteins [18]]. Reflecting the weak-field nature of its roughly tetrahedral iron coordination, the subdomain of iron–sulfur biochemistry gives a drastic further limitation to three basic forms: high-spin (*S* = 2) Fe(II), high-spin (*S* = 5/2) Fe(III), and S(−II). All iron–sulfur clusters are made out of these three building blocks only. However, coordination-chemical variability is regained in cluster formation, in direct ligation by occasional amino acid side chains other than Cys, and in networks of hydrogen bonds.

A very rich range of magnetic properties ensues from the clustering of iron ions affording spin ladders with system (or: cluster) spin states running from *S* = 0 all the way up to the sum of spins of all individual iron ions. For example, a 4Fe cluster in its physiologically common reduced state [4Fe–4S]^+^ formally has 3 Fe(II) and 1 Fe(III), which means that the system spin can range from *S* = 1/2 to *S* = 2 + 2 + 2 + 5/2 = 17/2 (a putative *S* = 11/2 form has recently been reported [19]). The quantum–mechanical determinant here is the resultant of opposing interactions: exchange coupling inducing antiparallel coupling of individual spins versus double exchange inducing parallel coupling of individual spins. Qualitatively this balance is related to degree of electron delocalization and to cluster structure, although after well over half a century of research on double resonance, there is still much to be desired in terms of quantitative relation with structure ([20] and refs quoted therein).

From the vantage point of the EPR spectroscopist interested in complex biological iron–sulfur systems this presents a flag for caution: any system other than one with a well isolated *S* = 1/2 ground state is likely to compromise our basic quest for concentration sensitivity. Fortunately, the majority of iron–sulfur clusters in proteins thus far reported on has an *S* = 1/2 ground state in one of its oxidation states, and is, therefore, expected to be detectable by System-EPR in complex systems. The most conspicuous example of this is the chain of respiratory complexes of many species in which usually all EPR-detectable iron–sulfur clusters have *S* = 1/2 and thus, typically, the majority is readily detectable even in human whole-tissue samples [21, 22].

## System-EPR and the sensitivity problem

Absorption of microwaves in EPR spectroscopy relies on differential population of magnetic sublevels induced by the Boltzmann distribution of states. For a two-level system (i.e., *S* = 1/2) the ratio of molecules in the ground state, *n*_0_, over that in the excited state, *n*_1_, is *n*_0_/*n*_1_ = 1/(e^−Δ*E*/*kT*^) in which *k* = 0.695 cm^−1^ K^−1^ is the Boltzmann constant, *T* is the absolute temperature in Kelvin, and ∆*E* is the difference in energy between the two states. For resonance to occur ∆*E* should equal the microwave energy, which in an X-band spectrometer, operating at circa 9.50 GHz, is *hv* ≈ 0.317 cm^−1^. At ambient temperature this gives a very small fractional surplus of molecules detected by EPR of *n*_0_/*n*_1_ = 1.002, which only increases to 1.036 at *T* = 13 K. This latter temperature value is often used in X-band EPR studies of iron–sulfur protein as a compromise for the detection of 2Fe, 3Fe, and 4Fe clusters at a single temperature, since it is the approximate limit above which the signal of fast relaxing [3Fe–4S]^+^ clusters will start to deform due to lifetime broadening, and it is also the approximate limit below which it becomes practically impossible to measure the signal of slow relaxing [2Fe–2S]^+^ clusters under non-saturating conditions.

A practical translation of this back-of-the-envelope calculation is that iron–sulfur proteins with *S* = 1/2 signals under optimal conditions have a concentration detection limit of the order of 0.1–1 μM in an X-band spectrometer (and a higher limit in any other spectrometer); recording of high-quality spectra would typically require at least an order-of-magnitude higher concentration. As a practical rule of thumb these numbers may have to be increased by 1–2 orders-of-magnitude for high-spin systems and by another 1–2 orders-of-magnitude for integer-spin systems. For System-EPR in whole cells this means that even *S* = 1/2 clusters may be at or below the detection limit unless the protein under study occurs naturally in relatively high concentration, such as the respiratory chain complexes in heart tissue [21]. With other proteins it is usually necessary to construct overexpression strains. This extra molecular-biological effort should, furthermore, be combined with efforts to optimize signal-to-noise ratio by chemical and physical means.

EPR spectroscopy is not limited to X-band. Spectrometers that run at different frequencies have been build ranging from sub-GHz radio waves up to near THz far infra-red waves. In addition, a variety of double resonance and pulse techniques have been introduced. In spite of the conceptual applicability of these machines it is important to realize that for *S* = 1/2 iron–sulfur proteins any move away from the standard continuous-wave X-band spectrometer will generally lead to a deterioration of the concentration detection limit, and is, therefore, equally generally not a good idea if one’s goal is a System-EPR study.

The outline of an ideal System-EPR experiment on, say, protein-x then is proposed to be as follows. A construct is made that overexpresses protein-x to a level that is readily detectable with X-band EPR (say 10 μM for bacterial of archaeal cells; eukaryots are more challenging in this respect) but that is at the same time not so high as to interfere with cell metabolism to the extent of compromising the original goal of the experiment i.e., monitoring protein-x under the physiological response of some actuator. In iron–sulfur biochemistry the latter is typically a stress factor, such as H_2_O_2_, or a change in concentration (e.g., by structural-gene deletion) of a protein involved in cluster biosynthesis or repair (see below). Wild-type cells should also be available for controls. For example, an EPR study of wild-type cells will establish a null-level of protein-x, but it may also indicate that the used growth conditions lead to background signals that are likely to interfere with EPR studies of overproducing strains. Purified protein-x should preferably also be available to optimize EPR machine settings. Finite spin–lattice relaxation rates of paramagnets translate into an optimal value for the microwave power at a given detection temperature for a given protein-x [23]. Below this value signal-to-noise is suboptimal, and above this value the spectrum increasingly deforms by saturation so that its amplitude is no longer proportional to chemical concentration and its shape cannot be analysed any more with extant simulators. EPR of purified proteins should always be reported at the optimal power level. However, when the quest for signal intensity becomes all dominating, e.g., in System-EPR studies of whole cells overexpressing protein-x, then it may be practical to allow for some level of saturation to make the signal detectable at all. (Semi-) quantitative analysis should then be linked to the saturation behaviour of protein-x as determined on the purified protein. A similar argument can be made with respect to over-modulation. EPR spectrometers use phase-sensitive detection with a high-frequency (typically 100 kHz) magnetic field of low amplitude (order of magnitude: 10 gauss for Fe/S spectra) as the modulator. Higher modulation amplitudes give higher signal amplitudes, but if the modulation amplitude exceeds a certain percentage (circa 50%) of the EPR line width, then the EPR signal deforms and its amplitude is no longer linear in the concentration. Again, EPR of purified proteins should be reported under optimal modulation, which is as high as possible without deforming the spectrum. In System-EPR one can consider to apply some over-modulation to gain signal intensity, but the modulation characteristics of protein-x should have been determined on the purified protein beforehand.

In what follows I will go through a number of reported examples of System-EPR studies on iron–sulfur proteins, in which I compare results from different authors, and check in how far the results fit with the ideal picture that I just drew. We will also come across inconsistencies and ambiguities, for example, related to unconvincing determination of the cluster redox state or cluster conversion state, and all this together will eventually lead to the formulation of a series of recommendations through which, it is hoped, System-EPR can acquire the important position that it potentially hold for future biochemical and medical research into the intrinsic cellular system of iron–sulfur proteins.

## Case study 1: the NEET protein

The protein that we now know under the name NEET (i.e., containing the amino-acid sequence N-E-E-T) was originally discovered as an EPR signal in a complex preparation, namely in mitochondrial outer membrane particles from rat liver [24] (see also [25–27]), and was only many years later heterologously expressed, purified and biochemically characterized in the form of a soluble recombinant fragment (reviewed, e.g., in [16]). It is interesting to compare the development of complex-system EPR versus single-compound EPR of this protein.

As a biochemical description, in brief, the protein is bound to the outer membrane of mitochondria through its N-terminal sequence. Recombinant expression is typically without this sequence, e.g., human mitoNEET (i.e., mitochondrial NEET) is used in the biochemical literature as a label for a heterologously expressed protein (actually three paralogs: mitoNEET, Miner1, and Miner2, in which Miner is an abbreviation of mitoNEET related [28, 29]) that misses the first 32 amino acids. The remainder sequence contains an unusual binding motif with 3 Cys and 1 His to bind a single [2Fe–2S]^(2+;+)^ cluster. The proteins mitoNEET and Miner1 occur as dimers with a twofold symmetry axis [30–32], and EPR shows magnetic-dipolar interaction between the two clusters in the dimer [33]. Miner2 is a monomer with two Cys_3_His_1_ binding motifs, each coordinating a [2Fe–2S] cluster [34]. The physiological function, or functions, of NEET is presently not clear; a plethora of possibilities has been proposed in recent years, usually related to Fe–S cluster transfer and/or modification [16, 35, 36]. Human NEET proteins have also been associated with a range of diseases most particularly as target for type-2 diabetes drugs [37, 38] or as chemotherapeutic target in breast cancer [39].

**Fig. 1:**
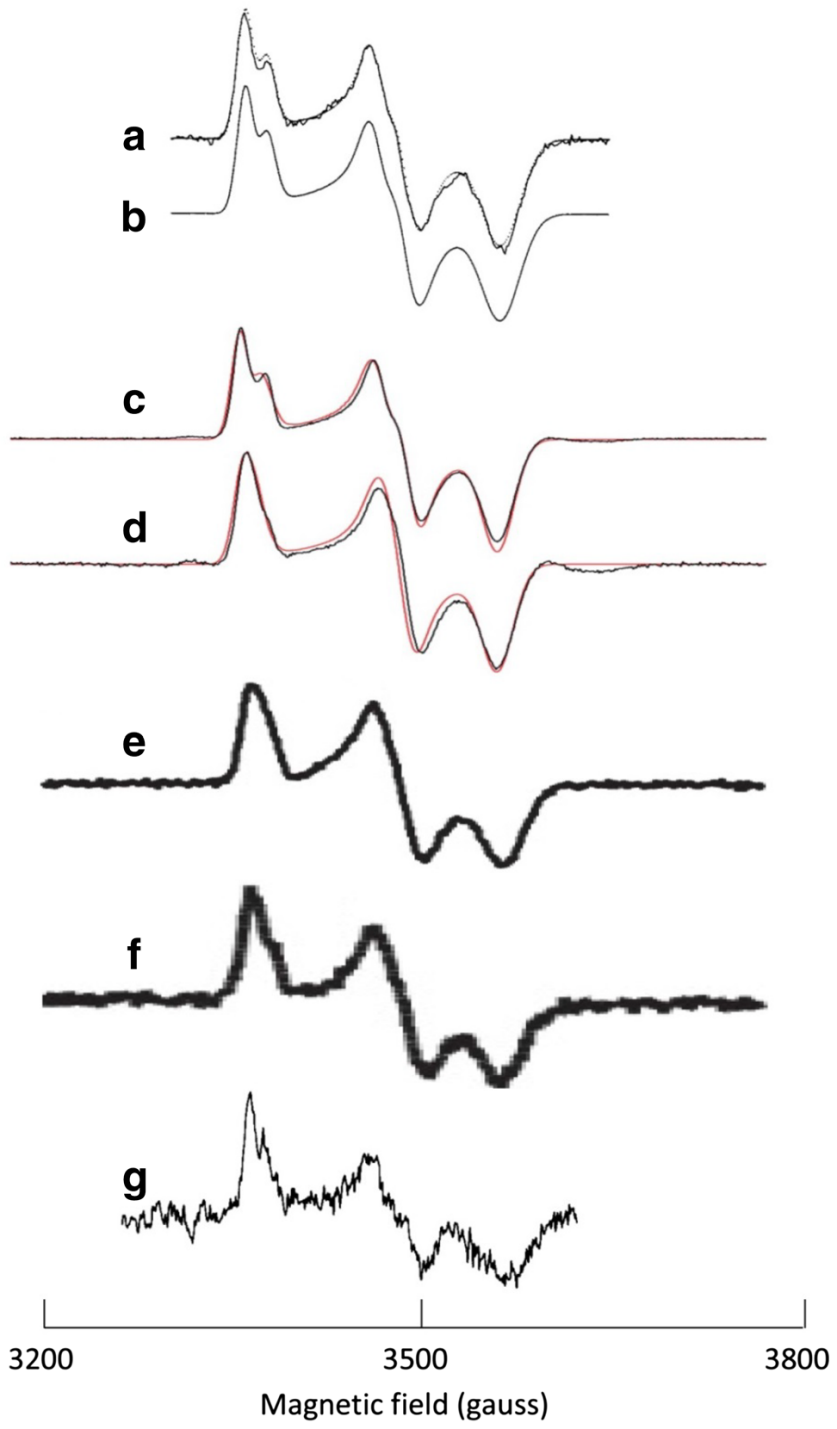
System-EPR spectroscopy of the Cys_3_His_1_[2Fe–2S]^+^ cluster in homodimeric mitochondrial outer-membrane protein mitoNEET. **a** spectrum of outer-membrane particles from bovine heart mitochondria reduced with NADH/dithionite taken at 93 K and 26 mW microwave power; (**b**, and dotted line in **a**) simulation assuming two different, non-interacting *S* = 1/2 systems in a 2:1 concentration ratio; **c** spectrum (black) and simulation (red) of purified recombinant (*E. coli* expressed) human mitoNEET soluble fragment fully reduced with dithionite taken at 20 K and 10 μW power; the simulation is based on two identical *S* = 1/2 systems with dipolar interaction; **d** as **c** but now partially reduced with dithiothreitol; **e** spectrum of purified recombinant (*E. coli* expressed) human mitoNEET soluble fragment fully reduced with dithionite taken at 20 K and 10 mW power; **f** as **e** but now in whole *E. coli* cells highly expressing the mitoNEET; **g** difference spectrum of dithionite-reduced bovine heart sub-mitochondrial particles, before and after treatment with lipoxygenase from rabbit reticulocyte, taken at 49 K and 2.2 mW power. Original spectra have been transposed to a microwave frequency of circa 9.50 GHz. Sources of data: (**a**, **b**) reproduced with permission from fig. 5 of [26]; (**c**, **d**) reproduced with permission from fig. 2 of [33] copyright © 2010 American Chemical Society; (**e**, **f**) reproduced with permission from part of fig. 1 and part of fig. 2 in [41] copyright © 2014 by the Americal Society for Biochemistry and Molecular Biology; **g** reproduced from part of fig. 2 in [27] copyright (1981) with permission from Elsevier

Figure 1 takes us through a time span of four decades of System-EPR on NEET protein. All spectra shown were taken in X-band at a microwave frequency of circa 9.2–9.5 GHz. The top traces A, B are System-EPR taken in 1978 at a temperature of 90 K from outer-membrane particles purified from bovine heart mitochondria [26]. The solid trace in A is the experimental spectrum; the dotted trace in A is identical to the solid trace B and is a simulated spectrum. Note the relatively high quality of the data when compared to the black trace C, which is from highly purified soluble fragment human mitoNEET taken at a temperature of 20 K in 2010 [33]. The original simulation in trace B was based on the assumption of two different *S* = 1/2 clusters, without interaction, in a concentration ratio of 2:1 [26]. We now know that this model cannot possibly be correct, since the NEET protein is a homodimer carrying two identical clusters [30–32]. That the splittings in the *g*_*z*_ an *g*_*y*_ feature of the X-band spectrum are actually reflecting magnetic-dipolar interaction between the two clusters, was unequivocally proven by the observation that these splittings in the EPR of purified protein become undetectable when the spectrum is recorded in Ka-band at a microwave frequency of 31 GHz [33]. Dipolar interaction is independent of the frequency and, therefore, becomes relatively less important compared to the electron Zeeman interaction with increasing frequency [40]. Furthermore, the red trace in C is a simulation under the model of two identical *S* = 1/2 clusters subject to mutual dipolar interaction [33].

The inter-cluster distance in the monomeric Miner2, with two [2Fe–2S], is very similar to that in the dimeric motoNEET or the Miner1. Therefore, dipolar interaction can be expected to be similarly important in Miner2. Remarkably, in a very recent analysis of the EPR spectrum of Miner2 this aspect has been ignored [29], although the shortest Fe–Fe distance between clusters in Miner2 of 13.6 Å (3TBN.pdb [34]) is less than that of 14.4 Å in mitoNEET (2QD0.pdb [31]).

A striking feature that emerges from a comparison of trace A versus trace C is their essential identity. Arguably the most important conclusion that one can draw from this observation is that the environment of the [2Fe–2S]^+^ cluster in the purified recombinant soluble fragment and in the complete protein embedded in its natural membrane is invariant, which gives confidence in the assumption that biochemical studies on the pure protein, for example binding of type-2 diabetes drugs, are quantitatively transferable to the ex vivo (and, by implication, to the in vivo) situation. The observed identity implies that mitoNEET in the membrane can be fully reduced, otherwise it would not exhibit the full splitting. The spectrum in trace D is from 50%-reduced, pure protein, which is in a statistical distribution of 25% oxidized, 50% with only one (or the other) cluster reduced, and 25% fully reduced. The unsplit pattern from single-cluster reduced molecules dominates [33].

With all this knowledge available it would appear that one is now in a good position to make the next step and study mitoNEET in whole cells by means of EPR monitoring. The data in trace E and F exemplify a first attempt in this direction: E is pure human mitoNEET, F is from whole *E. coli* cells expressing the human protein [41]. From an EPR spectroscopist’s point of view these results are disappointing. The data suggest that the signal-to-noise ratio for pure protein is comparable to that for whole-cell protein, which would only be realistic in case of an extremely high level of expression. Furthermore, the spectrum of fully dithionite reduced pure protein does not show the splittings from dipolar interaction. There appears to be some splitting in the whole-cell spectrum, but with a graphic line thickness comparable to that of the dipolar splitting, a conclusion is hard to draw. Comparing the former study (trace C, D) with the more recent one (trace E, F) we note that the measuring temperature in both cases was 20 K, but that the microwave power used in the latter is three orders of magnitude higher (10,000 μW) than in the former (10 μW). An unwritten law of EPR spectroscopy states that if one uses a single power level without providing power plots (i.e., plots of normalized amplitude versus power level at a fixed temperature) the implication is that the used power level has actually been determined to be optimal (i.e., as high as possible without significant saturation) (cf [23]). The absence of dipolar splitting in traces E and F may well be the result of spectral deformation by heavy saturation. We have argued, above, that purposeful saturation may be a legitimate means of increasing signal-to-noise levels to practical values for System-EPR studies, however, the details of the extent of saturation should be documented. In summary, NEET proteins can be studied by EPR as pure proteins, in subcellular fractions, and in whole cells, but exploration of the latter two areas has still a long way to go.

Finally, as an illustration of the potential of System-EPR we recall here an old, and apparently forgotten experiment on mitoNEET measured in sub-mitochondrial particles. Spectra of these systems are overwhelmed by signals from the respiratory chain, and so it is not altogether trivial to detect the signal of mitoNEET at natural expression levels against that background. Nevertheless, trace G in Fig. 1 is undoubtedly the EPR fingerprint of the protein. It was obtained as a difference spectrum of untreated particles minus particles treated with reticulocyte lipoxygenase [27]. It appears that the latter treatment destroys the iron–sulfur cluster of mitoNEET, which is an observation with putative mechanistic and medical implications.

## Case study 2: repair and synthesis of mainstream enzymes

**Fig. 2:**
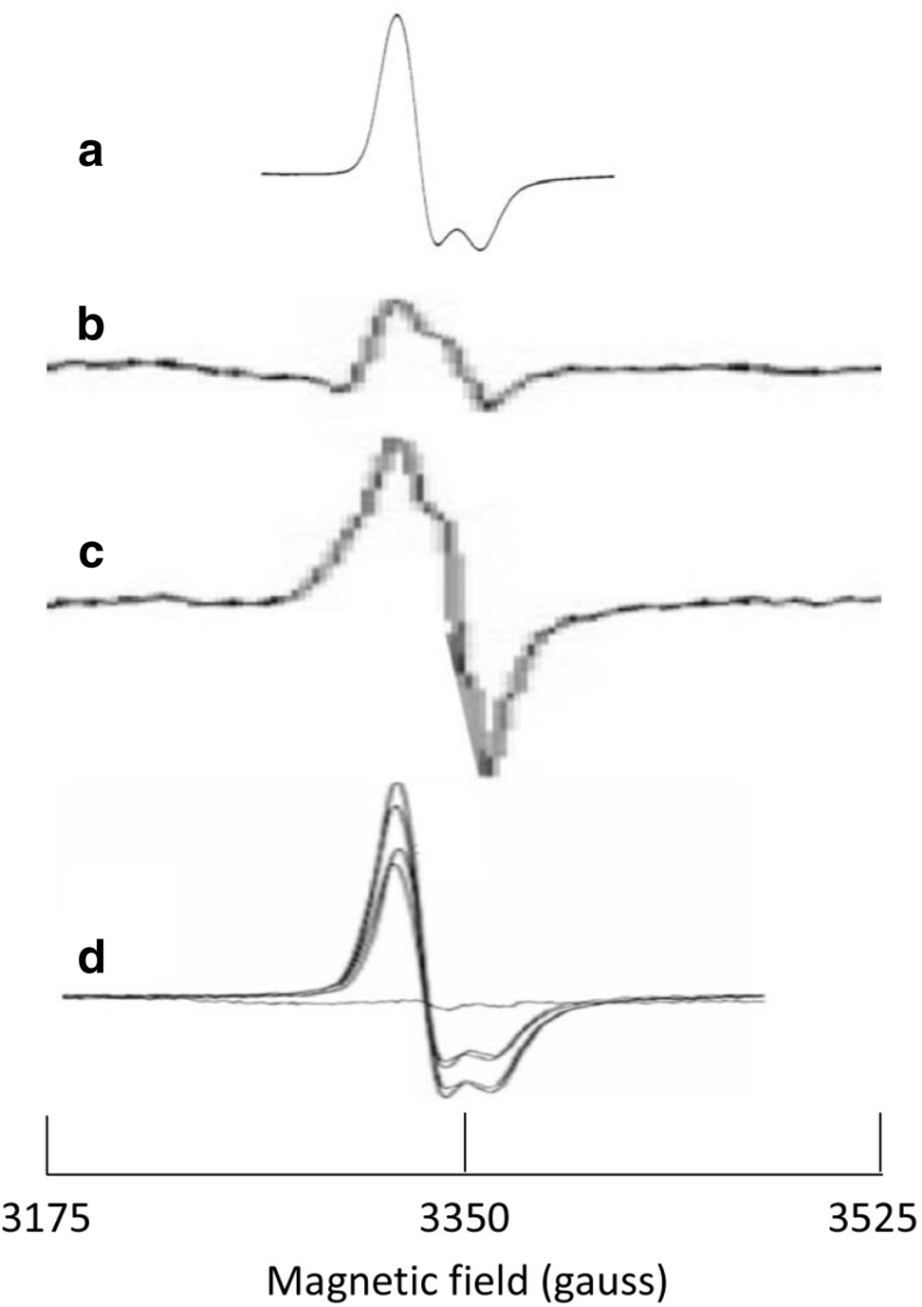
System-EPR spectroscopy of the [3Fe–4S]^+^ cluster in *E. coli* fumarase A. **a** spectrum of purified enzyme in its oxidized, inactive form; **b** spectrum of aerobically grown, fumarase A overexpressing *E. coli* cells, subjected to H_2_O_2_, taken at 10 K and 2.4 mW power; **c** as **b** but now for cells that lack the *yftE* gene for putative Fe/S cluster repair protein YftE or RIC; **d** spectra of anaerobically grown, fumarase A overexpressing *E. coli* cells that lack the genes for catalases, NADH peroxidase, and the Suf operon, taken at 15 K and 1 mW power. Decreasing amplitude in **d** corresponds to incubation with H_2_O_2_ for 1, 6, 10, 3, and 0 min. Original spectra have been transposed to a microwave frequency of circa 9.45 GHz. Sources of data: **a** reproduced with permission from fig. 4 of [43] copyright © 1992, American Chemical Society; (**b**, **c**) reproduced with permission from part of fig. 2 in [44] copyright © 2007 by the American Society for Biochemistry and Molecular Biology; **d** reproduced with permission from part of fig. 5 in [47] © 2010 Blackwell Publishing Ltd

The iron–sulfur cluster biosynthetic machinery can not only be studied with System-EPR by looking at its components in action in the cell, but also from a different perspective by following its effects on the integrity and reactivity of catalytic Fe–S clusters in household enzymes in the central pathways of metabolism. In this respect, the single [4Fe–4S]^2+^ cluster carrying citric-acid cycle hydro-lyases aconitase and fumarase have come to enjoy some interest, which may be related to the fact that, when inactivated by loss of a single iron ion, the enzymes are left with a [3Fe–4S]^(+;0)^ cluster whose oxidized form happens to be relatively easily detectable by EPR because of its near isotropic *g* tensor and because other Fe–S clusters typically are EPR silent in their oxidized state. *E. coli* produces three fumarases: A, B, and C: fumarase A and B share 90% amino-acid sequence identity and contain a single redox-invariant [4Fe–4S]^2+^ cluster with three Cys ligands for three iron ions and with one iron ‘coordinatively unsaturated’, i.e., free to accommodate the substrate fumarate for hydration to malate. Fumarase A and B are differentially expressed (aerobic versus anaerobic growth), but the physiological relevance of this is not clear: the two enzymes are very similar in their biochemical and biophysical properties [42]. Fumarase C is genetically unrelated and does not contain any prosthetic group. With reference to Fig. 2 we take the case of fumarase-A repair and synthesis as an example of System-EPR.

Trace A reproduces the EPR spectrum by Flint et al. of purified *E. coli* fumarase A in its oxidized, inactive form [43]. Trace B is the spectrum by Justino et al. of aerobically grown, fumarase A overexpressing *E. coli* cells subjected for 5 min to 4 mM H_2_O_2_ [44]. Trace C is from similarly treated cells in which the gene *yftE* has been deleted. The protein YftE (recently proposed to be renamed as RIC for ‘repair of iron centers’ [45]) carries a dinuclear iron oxo cluster and has been implicated in repair of Fe–S clusters ([46] and refs quoted therein). The circa threefold higher amplitude of the [3Fe–4S]^+^ signal in trace C versus B was interpreted as an indication that in the Δ*yftE* cells fumarase A was less well protected against oxidative damage by H_2_O_2_ [44]. Why is the shape of the spectra in the whole-cell preparations so different in detail from that of the purified enzyme in trace A? The authors also provide a baseline spectrum of wild-type cells not overexpressing fumarase A (fig. 2 in [44]). The baseline exhibits a signal of unidentified origin with an amplitude that is comparable to that of the overexpressed fumarase 3Fe signal. Since the traces in B and C are corrected for this baseline, it may be that the details of the 3Fe signal have suffered in the subtraction procedure.

Should we then conclude to have reached a limit of spectral resolution of System-EPR spectroscopy on iron–sulfur proteins? Jang and Imlay carried out a comparable experiment on H_2_O_2_ incubation of fumarase A overproducing, anaerobically grown *E. coli* cell that lack the genes for catalases and NADH peroxidase (denoted Hpx^−^ cells) and the Suf operon (Δsuf). The resulting EPR spectra after incubation with 0.2 mM H_2_O_2_ are reproduced in trace D. For a detailed biochemical interpretation of this experiment the reader is referred to the original work [47]. The point to make here is that the quality of the EPR spectra (trace D) is almost comparable to that of the purified enzyme (trace A). This quality difference with the data in traces B and C is not due to a major difference in EPR operation conditions; if anything, the settings used for trace D (15 K and 1 mW) are possibly a bit suboptimal compared to those used for trace B and C (10 K, 2.4 mW). Overexpression levels of fumarase A may have been significantly higher in the experiment of trace D compared to that of traces B and C; the original publications [44, 47] do not provide data that would allow for a quantitative comparison of expression levels. Another difference is that of aerobic (traces B and C) versus anaerobic (trace D) growth, since only the former leads to the background signal. Thus, a general conclusion that one can draw from these experiments is that in System-EPR the background signal (or rather its absence) may be an equally important optimization object for the quality and analyzability of the data as the foreground signal.

Similar System-EPR experiments have been carried out on aconitase; see, e.g., [44, 48–50].

## Case study 3: global versus specific regulation

The homodimeric SoxR (superoxide response) protein is a bacterial regulator of expression of a limited number of genes in bacteria such as Gram-negative *Pseudomonas aeruginosa* (*Pa*SoxR) or Gram-positive *Streptomyces coelicolor* (*Sc*SoxR). Each subunit carries a conventional [2Fe–2S]^(2+;+)^ cluster that acts as a redox sensor, e.g., of oxidative stress. Only enteric bacteria, such as *E. coli*, carry the complete *soxRS* regulon; *Ec*SoxR directly activates global regulator *Ec*SoxS that controls transcription of over a hundred genes (cf [51, 52] and refs quoted therein). EPR studies on SoxR, described below, were typically aimed at relating FeS cluster redox state to physiological functioning, but we will focus here on evaluating the quality of their System-EPR approach.

The reduced form of the purified *E. coli* protein exhibits a near-axial spectrum with *g* values 2.007, 1.922, 1.903 [53]. In this study, saturation characteristics were not specified. As a rare event in System-EPR Gaudu et al. carried out a study of SoxR in an *E. coli* strain in which overproduction was deliberately tuned: “We used mild inducing conditions with the goal of producing just enough SoxR to give a clear EPR signal without overwhelming either the cell’s capacity to produce other proteins or its ability to reduce SoxR” [54]. Also of note, in this study is the rigorous quantification of the EPR signal’s response to redox cycling by reference to the maximal EPR intensitity of SoxR observed in cells after lysis with lysozyme plus DNase plus protease inhibitor, followed by full reduction with dithionite. This absolute concentration of SoxR was, furthermore, checked versus the increase in iron content of overexpressing cells versus plasmid-free cells of the same density, and the iron number was found to agree within a few percent with the EPR integration. These quantifications were done on top of the more common, and less accurate densitometric quantification of SDS-PAGE bands with purified SoxR as the standard [54]. Unfortunately, also in this study EPR saturation properties were not documented.

**Fig. 3:**
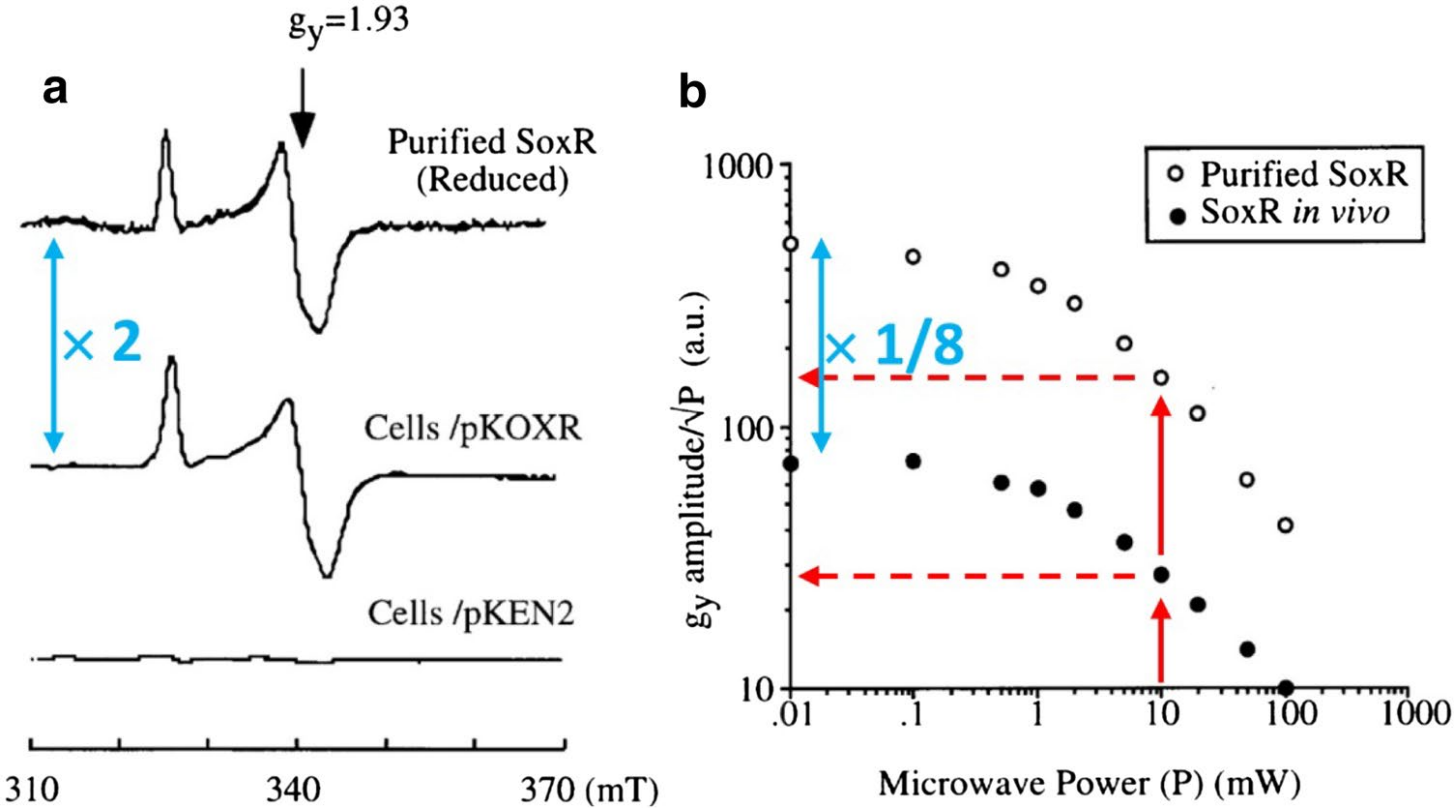
System-EPR spectroscopy of the [2Fe–2S]^+^ cluster in homodimeric *E. coli* regulatory SoxR (superoxide response) protein taken at 30 K and 10 mW power at 9.47 GHz. The figure has been reproduced from fig. 1 in [55] © (1997) by The National Academy of Sciences. pKOXR and pKEN2 are SoxR overexpressing and control constructs, respectively [56]. The colored symbols have been added by the present reviewer. The blue symbols indicate a discrepancy of a factor of 16 in electronic amplification factor between the EPR data of panel **a** versus panel **b**. The red symbols in the power plots indicate that the spectra in panel **a** have been taken under conditions of more than 50% saturation

Only the densitometric concentration reference was used in a subsequent System-EPR study by Ding and Demple [55]. Here, saturation was measured both on the whole-cell signal and on the purified protein as a verification (in addition to signal shape equality) of the identity of the signal from SoxR-overproducing cells. A few remarks are in order on these saturation plots (Fig. 3). Firstly, the EPR amplitude from cells (18 μM) is almost twice that of the purified SoxR (10 μM), however, the unsaturated data points in the power plots give an EPR cell signal amplitude that is over 8 times less than that of the pure protein. Furthermore, all EPR spectra are reported to be taken at a microwave power level of 10 mW. According to the cited power plot this value implies reduction of the EPR amplitude by saturation to less than 45% of the unsaturated value. This would create an inconsistency with the claim that EPR quantification (18 μM), when compared to densitometric quantification (20 μM), shows that SoxR is 90% reduced in resting cells. Quantification of heavily saturated EPR signals is not common, and would at least require documented correction for the saturation level. Such problems attest to the necessity that System-EPR studies are accompanied by rigorous documentation of EPR properties including quantification, (over-) modulation, power saturation, and redox state.

**Fig. 4:**
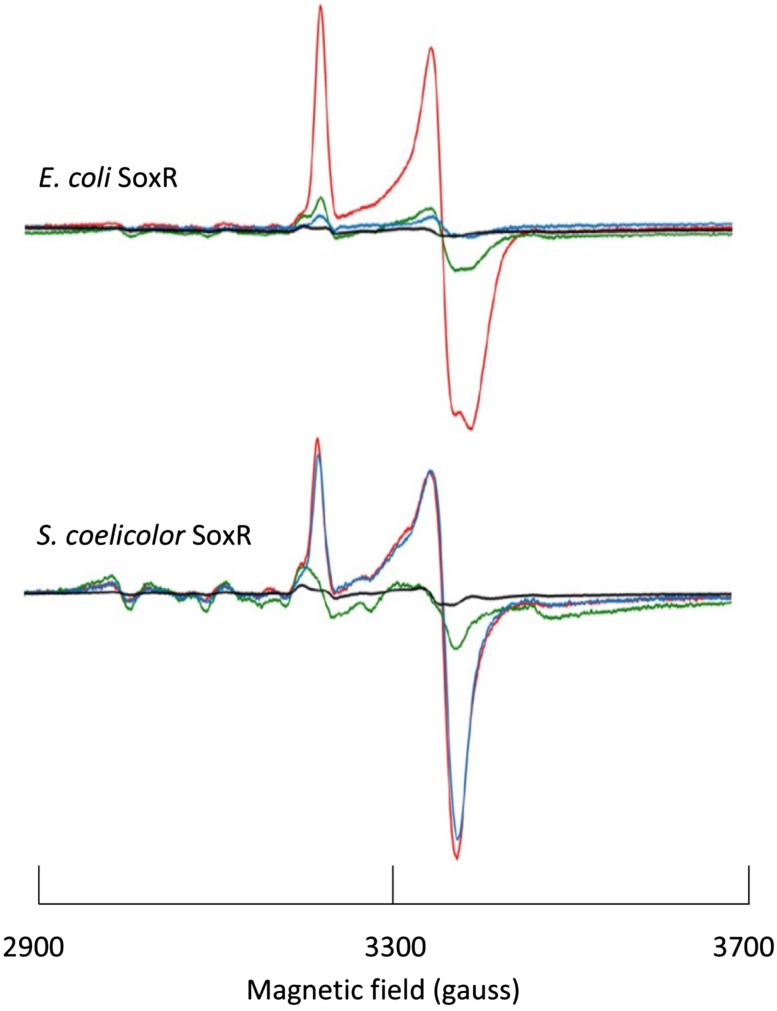
System-EPR spectroscopy of the [2Fe–2S]^+^ cluster in two SoxR proteins. *E. coli* and *S. coelicolor* SoxR were each expressed in *E. coli* presumably to the same level. Note, however, the difference in Mn(II) background signal intensities, e.g., at fields below 3185 gauss. The EPR spectra were taken at 15 K and 1 mW power at 9.05 GHz. Color coding of traces: no addition (red), phenazine methosulfate added (green), methyl viologen added (blue), vector control (black). The data have been reproduced with permission from part of fig. 7 in [57] © 2013 John Wiley & Sons Ltd

In a more recent study, Singh et al. compared SoxR in whole cells from enteric versus non-enteric origin (i.e., with versus without SoxS). The study attempted to relate differential redox properties to differential physiological functioning [57]. For example (Fig. 4) the EPR signal from both *E. coli* and *S. coelicolor* SoxR, overexpressed in *E. coli* cells, disappeared (i.e., oxidation of the [2Fe–2S] cluster) upon incubation with high-potential oxidant phenazine methosulfate (PMS). On the other hand, the low-potential oxidant methyl viologen (MV) only afforded oxidation of the cluster in *E. coli* SoxR, suggesting a differential response to oxidative stress, which may be related to a difference in cluster reduction potential [57]. Although the amplitudes of the two SoxRs in Fig. 4 are similar (the ratio of second integrals of the bottom versus top spectrum is circa 0.8), the background signals and noise levels differ by a factor of circa three. With equal cell density levels this discrepancy of more than a factor of three indicates different expression levels and/or different degrees of reduction. The authors note that ‘The expression level of SoxR in the soluble fraction of cells subjected to EPR analysis was confirmed on SDS-PAGE in a parallel experiment’. Unfortunately, no specific data are given.

## Recommendations

From the very instant (1960) of the discovery of iron–sulfur proteins onwards EPR spectroscopy has been applied in parallel to purified proteins and to more or less ‘intact’ systems such as membrane particles [1, 17]. By around 1980 System-EPR of iron–sulfur proteins had evolved into a quantitative technique based on the quality optimization of data via experimental key parameters, notably, microwave intensity, sample temperature, field-modulation amplitude, spin counting by spectral simulation, and statistics of multiple preparations (e.g., [58, 59]). The subsequent arrival of recombinant methodology for homologous overexpression or high-level heterologous expression of protein structural genes opened up the System-EPR approach beyond applicability to abundant respiratory-chain complexes only. In early studies on overexpressing cells due care was taken to calibrate redox levels, e.g., by strict anaerobic handling of samples after anaerobic growth [60] or by dithionite-reduction of permeabilized cells [54]. On the other hand, other basic characteristics, e.g., expression levels [60] or power-saturation data [54] were not always explicitly documented.

Entering the twenty-first century iron–sulfur protein biochemistry has moved forward into the systems-biological field of FeS cluster homeostasis (biosynthesis, breakdown, and repair) with multiple ramifications in the medical sciences. Under this constellation, one would expect to see System-EPR to flourish as a method of choice to identify and monitor FeS sites of specific proteins in action under normal versus stressed conditions. Interestingly, the size of the harvest thus far does not strike one as impressive. Moreover, when checked against the quality criteria indicated above, more recent studies frequently leave something to be desired (cf. the case studies, above). The wish list, below, is offered as a coherent collection of suggestions to improve quality and thus impact of System-EPR.

### Construction of cell line

A recombinant cell line has to be constructed that expresses the protein-x to be monitored to concentrations that are conveniently detected by EPR. The detection limit for not too broad Fe/S *S* = 1/2 signals under optimized settings, with good baselines, and with significant averaging is very approximately > 0.1 μM, however, based on literature (e.g., the case studies above) a more realistic concentration to strive for would rather be of the order of 10 μM. Significantly higher concentrations will make the EPR spectroscopist happy, but may increasingly start to interfere with regular cell metabolism. Quantifying the relation between expression level and, e.g., growth rate, maximal optical density, housekeeping activities, etc., may be indicated, be it a laborious undertaking. From this perspective, homologous expression may be preferred although obviously will not always be possible.

Frequently, the protein focused on will be a monitor for some stress condition, in particular, the deletion of one or more catalyzing or regulatory components of the Fe/S biosynthetic machinery. Thus, other genes in the cell will have to be modified (e.g., ∆ strains). This construct without the modification in the expression of the protein-x to be EPR-monitored is the proper reference for expression levels and for EPR baselines, which implies that the change in expression of protein-x is the last modification to be implemented. Wild-type cells may be an acceptable substitute.

If orthologous proteins are studied in a single host, then basal expression of the intrinsic host protein should be genetically eliminated. Furthermore, there is no a priori reason to expect identical expression levels for orthologs, and this gives added relevance to quantification of these levels.

### Sample preparation

If documentation in the literature of protein properties (notably: EPR spectral line shapes, redox properties) is absent or incomplete, the protein should be purified to homogeneity from wild-type or increased-expression cells as a useful, if not indispensable, reference system for spectroscopy. When technical difficulties preclude the use of homologous expression systems (e.g., the expression of human mitoNEET in *E. coli*), possible implications of the system’s unnaturalness should be addressed.

The composition of culture media frequently stems from publications of the distant past, and the rationale behind specific concentration values is not always retraceable. Some ingredients may well be in high excess over their growth-limiting value, may end up unprocessed in the cell, and thus may contribute to an irrelevant but annoying EPR background. Mn(II) aspecifically bound to proteins produces an EPR spectrum that overlaps with *S* = 1/2 iron–sulfur spectra, and thus can interfere with spectral analysis. A case in point is the Mn(II) background signal in Fig. 4. Reducing Mn(II) salts to near growth-limiting values (if any) is a relatively simple, but from the EPR-spectroscopy viewpoint very rewarding action.

Strict anaerobic handling of samples may be indicated for two reasons. First, it may be a way to keep certain Fe/S clusters in their physiological reduced state following anaerobic cell growth. Second, even from aerobes Fe/S clusters are known to deteriorate due to oxygen sensitivity during sample handling. This may be particularly true following cell fractionation and purification of organelles or sub-organelle particles.

Low signal intensities may be fought by concentrating, e.g., whole cells to the max. The simplest approach is to re-suspend cells in minimal buffer volume. More thorough packing may be obtained by filling an EPR tube to, say, twice the measuring volume, and then placing the tube in a centrifuge, which may require the use of shorter than standard tubes. This approach knows different levels of sophistication: on the high side custom inserts may be built for particular rotors (cf [61]); at the poor man’s level it may suffice (for whole cells) to swing the EPR tube with a stretched arm. After centrifugation, the upper liquid may be decanted or pipetted away before freezing the sample. Relevant factors to consider are anaerobicity, reproducibility and accessibility for to-be-added dissolved reactants.

The latter is also a general point of concern: the target Fe/S protein may be enclosed in a membrane that is essentially impermeable for charged compounds such as the ionic, universal reductant dithionite or oxidant ferricyanide. To be able to bring the FeS cluster of a target protein fully in a particular reference redox state may require to make cells or other closed-membrane systems ‘leaky’. Different systems may require different approaches: in the absence of cryoprotectants a few liquid nitrogen freezing/thawing cycles may suffice for, e.g., vertebrate cells; Gram-negative bacterial cells may be treated with lysozyme (cf [54]). Sonication in the presence of reactant is another option.

### Analytical chemistry

A useful reference value is the total protein of over/high expression cells versus that of uninduced cells. Increase in iron content may in favourable cases be related to over/high expression of an iron–sulfur protein (cf [54]), however, there is a need for more literature data on this matter.

Expression levels can be determined from quantitative SDS-PAGE, activity measurements, Western blots, ELISA, and from EPR doubly-integrated amplitudes in defined redox states. Comparing the EPR integral to other expression-level determinations also affords an estimate of apo-protein levels.

### Spectrometer settings

Since the EPR signal amplitude increases with the sample-tube diameter, wide tubes (up to circa 5 mm outer diameter) with thin walls are indicated. The limits are set by the dimensions of the helium-flow cooling system and by the tube’s toughness.

The spectrometer to be employed is a standard, continuous-wave, X-band (circa 9–10 GHz) machine. Choice of the spin system is generally limited to *S* = 1/2. Helium-flow cooling is generally required for Fe/S clusters.

**Fig. 5:**
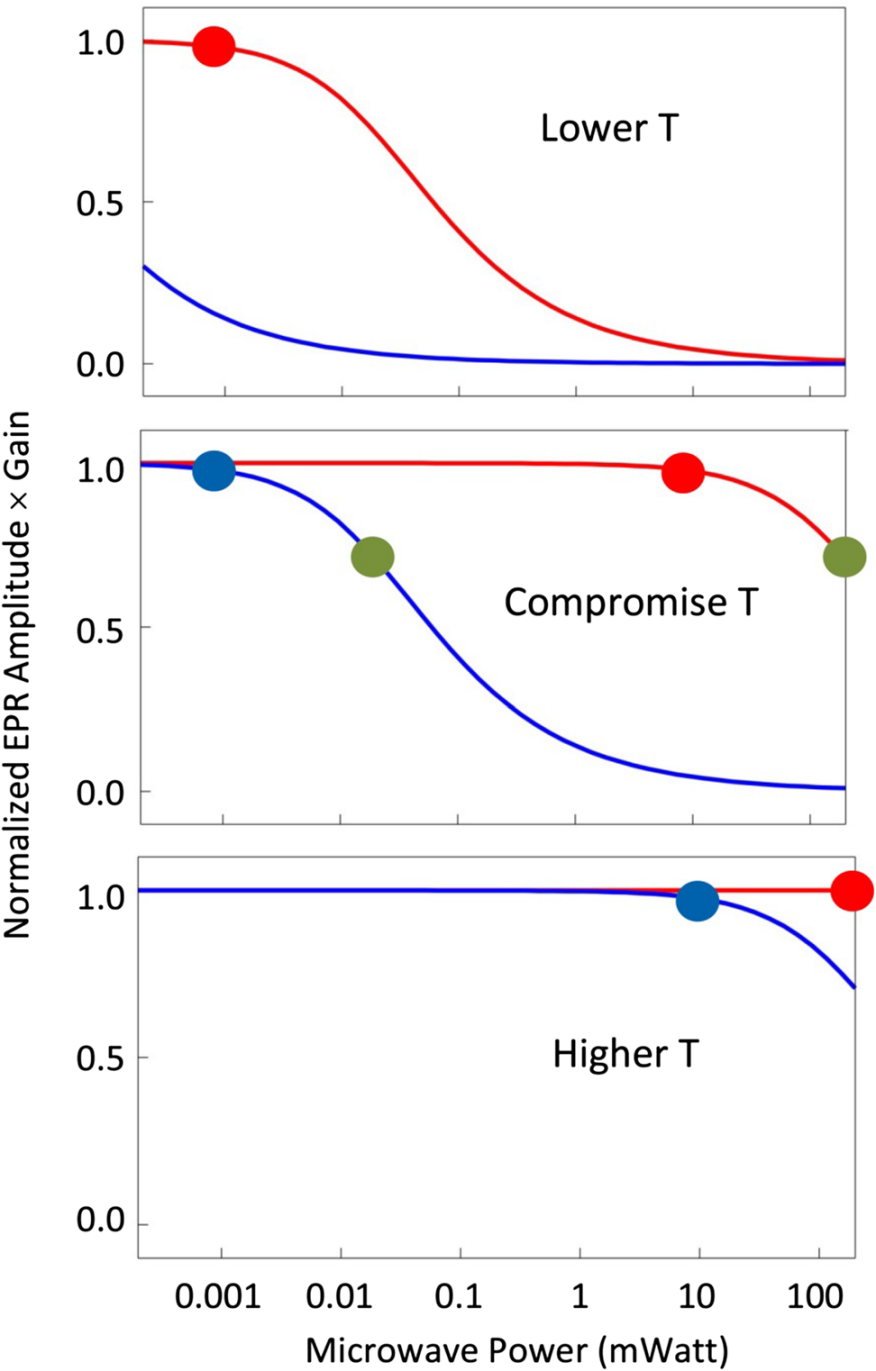
Schematic illustration of power saturation characteristics as a function of temperature for a slow-relaxing (blue) and a fast-relaxing (red) spin system. The semi-logarithmic plots are curves for spectra dominated by temperature-independent, inhomogeneous broadening, i.e., normalized amplitude = (1 + *P*/*P*_0.5_)^−0.5^, in which *P* is the power in milliwatt and *P*_0.5_ is the power at which the signal is 50% saturated (cf [62]). The middle panel is for a compromise temperature at which measurements of both systems can be made without saturation, be it sub-optimal for the slow-relaxing species. The blue and red dots are close-to ideal points, namely, at near-to-non-saturating conditions (i.e., 0.98 intensity, or 1.00 intensity when saturation is absent). The green dots indicate a typical System-EPR setting of ‘over’-optimization in which signals are measured under partial (here: 30%) saturation. At lower temperatures (top panel) the slow-relaxing species can only be measured under heavy saturation; at higher temperatures (bottom panel) detection of the fast-relaxing species is always sub-optimal

At a given microwave frequency (here: circa 9.5 GHz) a given spin system (here an Fe/S cluster in an *S* = 1/2 state) has an optimal set of EPR measuring conditions determined by its spin–lattice relaxation properties. For example, the sample temperature should be below the value at which lifetime broadening sets in (typically 20–25 K for a [4Fe–4S]^+^ cluster), but it should not be so low that the signal cannot be measured without saturation (typically [2Fe–2S]^+^ clusters cannot be reliably measured at a temperature as low as that of liquid helium, 4.2 K). At a given temperature there is an optimal microwave power for detection, namely, the highest power at which the signal is just not beginning to saturate (cf [23]). This value decreases with decreasing temperature. The optimal value should be within the spectrometer’s range of attainable power levels, which is typically from 200 nW (− 60 dB) to 200 mW (0 dB). If this is not the case, then the temperature should be changed (see the schematic illustration in Fig. 5).

Furthermore, spectrometers tend to become less stable at lower power setting (e.g., because the feedback electronics, that try to keep the source frequency equal to the loaded-cavity frequency, receive a weaker signal), but often also in a small range at the very highest power levels of the order of 100 mW and higher. If increasing signal-to-noise is of the essence, as it may be in System-EPR, minor loss in EPR resolution may be traded off against increased signal amplitude by partial saturation. If this approach is taken, then power-saturation characteristics must be determined, preferably on the purified protein-x, and must be documented. Omission of saturation documentation should imply that EPR signals have been recorded under optimal non-saturating conditions; unfortunately, in many publications (cf the case studies, above) this condition is not generally fulfilled.

EPR spectrometers use phase-sensitive detection to improve signal-to-noise ratios: a small, rapidly varying (100 kHz) magnetic field (known as the modulation field) is added to the ‘static’ slowly scanning field, and is in-phase detected and amplified. The result is the first derivative EPR signal whose amplitude is linear in the modulation amplitude under the condition that the latter is significantly less than the EPR line width. Normally, the optimal setting is a maximal modulation amplitude that just does not deform the EPR signal by overmodulation. If increasing signal-to-noise ratio is essential, as it may be in System-EPR, minor loss in EPR resolution may be traded off against increased signal amplitude by overmodulation (cf [63]). Overmodulation characteristics should be determined, preferably on purified protein-x, and should be documented.

### Spectroscopic analysis

Spin counting (i.e., the determination of the concentration of a spin system) in EPR is done by doubly integrating the first-derivative EPR spectrum to obtain the area under the EPR absorption spectrum, which may then be compared to the double integral of the spectrum of a standard of known concentration, e.g., a copper salt ([23]). In System-EPR this could be done by integrating the spectrum of purified protein-x, and comparing its amplitude to that of the protein-x spectrum from a complex system such as whole cells taken under identical spectrometer settings. If purified protein is not available and if signals from other proteins and/or from a poor baseline interfere, then it is usually advisable to simulate the spectrum of protein-x and to use the simulation for integration.

If several signals (from the same or from different proteins) are monitored, they may possibly be measured at a single, compromise temperature, but better quality data are obtained by measuring each signal at its own optimal temperature (cf Fig. 5). Furthermore, temperature can be used to discriminate overlapping signals by elimination. For example, many [2Fe–2S]^+^ signals are unbroadened at 90 K, while most [4Fe–4S]^+^ signals will be broadened beyond detection at this temperature.

The maximum intensity of an Fe/S signal in a complex system should be determined as a reference point after the cluster has been brought in a single redox state by reduction or oxidation. This usually requires making the complex system ‘leaky’ for reactant.

Comparison of this (doubly integrated) intensity with that of protein-x concentration (e.g., from SDS-PAGE) establishes the amount of apo and holo protein-x in the complex system.

If a cluster exhibits easy conversion to a different type, then the *t* = 0 situation should be determined by quantification, e.g., of fully reduced [4Fe–4S]^+^ versus fully oxidized [3Fe–4S]^+^.

In the early days of System-EPR it was quite common to report statistics of EPR quantification data from a series of independent preparations [58, 59]. In more recent studies only single-experiment data are reported; one can sometimes read statements like ‘representative data from X independent experiment’, or ‘the results are averages from X independent experiments’, but the full data are generally not given (also not in supplementary material) and it is not specified what an independent experiment is [41, 57] or how an EPR spectrum can be a representative of independent experiments [49]. Cell cultivation products can vary, and a statistical approach is laudable, but all data should be retrievable.

## Conclusion

Compared to the measurement by EPR spectroscopy of iron-sulfur clusters in purified proteins, their study in whole cells requires an added effort in experimental design. A general sensitivity problem inherent in the spectroscopy calls for specific strategies of increased protein expression and of scrutinous optimization of measuring conditions. Furthermore, the non-equilibrial nature of cell physiology dictates careful calibration of redox and cluster-conversion levels. Once these conditions have been fulfilled, the spectroscopy should afford specific and detailed information on Fe/S turnover that may be hard to get by any other method.
